# Patterns of brain structural alteration in COPD with different levels of pulmonary function impairment and its association with cognitive deficits

**DOI:** 10.1186/s12890-019-0955-y

**Published:** 2019-11-07

**Authors:** Minmin Yin, Haibao Wang, Xianwei Hu, Xiaoshu Li, Guanghe Fei, Yongqiang Yu

**Affiliations:** 1 Department of Radiology, The First Affiliated Hospital of Anhui Medical University, Hefei, 230022 Anhui China; 2 Department of Respiration, The First Affiliated Hospital of Anhui Medical University, Hefei, 230022 Anhui China

**Keywords:** Chronic obstructive pulmonary disease, Cognition, Magnetic resonance imaging, VBM, Diffusion tensor imaging

## Abstract

**Background:**

To explore patterns of brain structural alteration in chronic obstructive pulmonary disease (COPD) patients with different levels of lung function impairment and the associations of those patterns with cognitive functional deficits using voxel-based morphometry (VBM) and tract-based spatial statistics (TBSS) analyses based on high-resolution structural MRI and diffusion tensor imaging (DTI).

**Methods:**

A total of 115 right-handed participants (26 severe, 29 moderate, and 29 mild COPD patients and a comparison group of 31 individuals without COPD) completed tests of cognitive (Montreal Cognitive Assessment [MoCA]) and pulmonary function (forced expiratory volume in 1 s [FEV1]) and underwent MRI scanning. VBM and TBSS analyses were used to identify changes in grey matter density (GMD) and white matter (WM) integrity in COPD patients. In addition, correlation analyses between these imaging parameter changes and cognitive and pulmonary functional impairments were performed.

**Results:**

There was no significant difference in brain structure between the comparison groups and the mild COPD patients. Patients with moderate COPD had atrophy of the left middle frontal gyrus and right opercular part/triangular part of the inferior frontal gyrus, and WM changes were present mainly in the superior and posterior corona radiata, corpus callosum and cingulum. Patients with severe COPD exhibited the most extensive changes in GMD and WM. Some grey matter (GM) and WM changes were correlated with MoCA scores and FEV1.

**Conclusions:**

These findings suggest that patients with COPD exhibit progressive structural impairments in both the GM and the WM, along with impaired levels of lung function, highlighting the importance of early clinical interventions.

## Background

Chronic obstructive pulmonary disease (COPD) is a chronic progressive airflow restriction syndrome that is often accompanied by a variety of extrapulmonary complications. Central nervous system dysfunction is one such extrapulmonary complication [1]. Dag et al. [2] and López-Torres et al. [3] have shown that cognitive function is reduced in COPD patients; Yin et al. [4] found that cognitive impairment in COPD patients does not significantly differ by sex, region, educational level, smoking status, or alcohol drinking. The mechanism of COPD-related cognitive impairment may be associated with hypoxia-induced neurological damage [5], airway obstruction (measured by forced expiratory volume in 1 s [FEV1]) [6], and inflammatory mediators [7, 8]. However, studies on the topic have merely suggested an association rather than a causal link [9]; the mechanisms of brain pathology and cognitive impairment are likely to be complex and multi-factorial [10] and are not well understood.

To date, neuroimaging studies have found changes in brain structure, metabolism and function in patients with COPD. Ortapamuk et al. [11] found that blood flow perfusion in the frontal and parietal lobes of COPD patients was significantly reduced on SPECT. Previous studies have shown that the task and resting states of the prefrontal networks of COPD patients can be determined by functional magnetic resonance (fMRI) studies, and abnormal activation of multiple brain regions has been found [12, 13]. Zhang et al. found that the grey matter (GM) of COPD patients varied in many brain regions, such as the limbic and the paralimbic system [14]. In addition, diffuse injury was found in white matter (WM) in patients with stable COPD [15].

Previous neuroanatomical studies of COPD patients were based mainly on the classification of oxygen saturation [16]. The severity of the disease was stratified by oxygen saturation, the sample size was relatively small, and the range of damage in WM was reflected only by the fractional anisotropy (FA) value, which is not sufficiently comprehensive [15], thus introducing bias in the results. The Global Initiative for Chronic Obstructive Lung Disease (GOLD) guidelines recommend classifying COPD severity based on pulmonary function (measured by FEV1) [17] rather than oxygen saturation. In the present study, we used a larger sample size than previous studies and divided patients into multiple sub-groups based on lung function. The aim of this study was to explore patterns of brain structural alteration in COPD patients with different levels of lung function impairment and the associations of these patterns with cognitive function deficits. We hypothesized that COPD patients would exhibit different degrees of structural impairments in both GM and WM according to their levels of lung function and that these structural impairments would be correlated with cognitive functional deficits.

## Methods

### Subjects

A total of 115 right-handed individuals (26 severe, 29 moderate, and 29 mild COPD patients and a comparison group of 31 individuals without COPD) participated in the study. The comparison group consisted entirely of volunteers from the community, and the COPD patients were recruited from the Pulmonary Clinic and Inpatient Department of the First Affiliated Hospital of Anhui Medical University from March 2013 to December 2016. COPD was diagnosed and classified according to the 2013 GOLD guidelines [17]. The exclusion criteria were as follows: (1) other lung diseases; (2) comorbidities such as vascular complications of diabetes, liver failure, cardiovascular disease, neurologic disease, malignant tumours, obstructive sleep apnoea or other diseases known to affect cognition; (3) < 5 years of education; and (4) claustrophobia, ferromagnetic implants, or pacemakers [12, 15]. All participants underwent a complete physical examination administered by a respiratory physician and a neuropsychologist. All subjects provided written consent to participate and were informed of the possible risks of the study. The study was performed according to the principles outlined in the Declaration of Helsinki and was approved by the institutional ethics committee.

### Physiological and neuropsychological tests

The arterial partial pressure of oxygen (PaO_2_) and blood oxygen saturation (SaO_2_) were evaluated with a Stat Profile Critical Care Xpress system (Nova Biomedical, Waltham, MA, USA) within 24 h before MR scanning. The normal range for PaO_2_ was defined as PaO_2_>80 mmHg; mild hypoxia was defined as 60 mmHg<PaO_2_ ≤ 80 mmHg [18]. The normal range for SaO_2_ was defined as SaO_2_>94%; mild hypoxia was defined as 90% ≤ SaO_2_ ≤ 94% [19].

Patients underwent a standardized test of pulmonary function using a dry spirometer device within 24 h before MR scanning (Erich Jaeger GmbH, Hoechberg, Germany), 15 min after inhaling 400 μg of salbutamol (Ventolin; GlaxoSmithKline; London, UK); forced vital capacity (FVC), FEV1, and the FEV1/FVC ratio were recorded. For patients with FEV1/FVC ratios < 0.7, disease staging was predicted based on the FEV1% as follows: FEV1% ≥ 80% predicted mild COPD; 50% ≤ FEV1% < 80% predicted moderate COPD; and 30% ≤ FEV1% < 50% predicted severe COPD [17].

The MoCA was used to assess cognitive performance [20, 21]. Data were analysed with SPSS v16.0 (SPSS Inc., Chicago, IL, USA), followed by a test for homogeneity of variance. The means of the four groups were compared by one-way analysis of variance with a post hoc test to measure between-group differences. Statistical significance was set at *P* < 0.05.

### MRI data acquisition

All MRI examinations were performed on an MR750w 3.0 T MRI scanner with a 16-channel head coil (General Electric, Waukesha, WI, USA). We performed T1-weighted three-dimensional (3D) structural imaging, axial T2-weighted DTI spin-echo single-shot echo-planar imaging, and axial T2-weighted and fluid-attenuated inversion recovery imaging. Relevant parameters can be found in Additional file 1: supplementary materials.

However, as the presence of mild to moderate WM hyperintensities (WMHs) commonly accompanies ageing and neurodegenerative diseases [22], we graded the WMHs using the Fazekas scale [23] on the basis of visual assessment in both periventricular (0 = absent, 1 = caps or pencil lining, 2 = smooth halo, 3 = irregular periventricular hyperintensities extending into the deep WM) and subcortical areas (0 = absent, 1 = punctate foci, 2 = foci beginning to become confluent, 3 = large confluent areas). The total Fazekas score was calculated by adding the periventricular and subcortical scores together [24] and was regressed out in the following statistical models. Detailed information can be found in Table 1.

**Table 1:** Demographic and clinical characteristics of the participants

Variables	Comparison group (*n* = 31)	Mild COPD (*n* = 29)	Moderate COPD (n = 29)	Severe COPD (*n* = 26)	F/χ^2^ value	*P* value
Age	57.12 ± 8.66	59.38 ± 8.27	60.90 ± 10.63	63.42 ± 9.53	1.91	0.13^a^
Sex (M/F)	(22/9)	(14/15)	(13/16)	(18/8)	6.69	0.08^b^
Education level (year)	9.06 ± 2.13	9.28 ± 2.03	9.34 ± 1.97	9.19 ± 2.23	1.00	0.96^a^
BMI (kg/m^2^)	24.17 ± 2.93	24.08 ± 4.50	23.19 ± 4.27	22.19 ± 3.27	1.64	0.19^a^
Current smoking status (Y/N)	(17/14)	(11/18)	(14/15)	(16/10)	3.39	0.34^b^
Smoking index (packs/year)	22.26 ± 1.12	22.69 ± 1.42	23.03 ± 1.32	23.35 ± 2.54	2.28	0.08^a^
FEV_1_%	105.34 ± 21.61	85.18 ± 4.21*	65.93 ± 8.19**	38.16 ± 7.63***	143.51	0.000^a^
PaO_2_ (mmHg)	116.76 ± 6.64	99.75 ± 7.88*	77.18 ± 4.94**	66.50 ± 3.92***	393.71	0.000^a^
NormalPaO_2_, n(%)	31 (100)	29 (100)	9 (31.0)	0 (0)	89.14	0.000^b^
SaO_2_%	98.14 ± 0.43	96.23 ± 0.60*	94.56 ± 0.83**	91.67 ± 1.15***	342.28	0.000^a^
Normal SaO_2_, n(%)	31 (100)	29 (100)	21 (72.4)	0 (0)	87.18	0.000^b^
WMH score	1.64 ± 0.97	1.73 ± 0.81	1.69 ± 1.12	1.85 ± 1.09	1.13	0.94^a^

**Note**: Data are presented as the means ± standard deviations, except where otherwise indicated. **Abbreviations**: a, analysis of variance; b, χ^2^ test; BMI, body mass index; F, female; FEV1, forced expiratory volume in 1 s; M, male; N, no; PaO_2_, arterial partial pressure of oxygen; SaO_2_, oxygen saturation; WMH, white matter hyperintensity; and Y, yes. *Significantly different from controls (P < 0.001). **Significantly different from patients with mild COPD (*P* < 0.001). ***Significantly different from patients with moderate COPD (*P* < 0.001)

### VBM analysis

GM atrophy was assessed with modulated VBM using Statistical Parametric Mapping 12 (SPM12, http://www.fil.ion.ucl.ac.uk/spm/software/spm12) [25]. The 3D structural data were segmented into GM, WM, and cerebrospinal fluid using the software VBM12, and Procrustes-aligned GM images were generated by a rigid transformation. These components were normalized to the standard Montreal Neurological Institute space by affine and non-linear registrations and the diffeomorphic anatomical registration using exponentiated Lie algebra algorithm. SPM12 was used to smooth the images with an 8-mm Gaussian kernel. An F-test was used to initially identify GM areas differing among the four groups, and post hoc analyses were performed to search for pairwise differences between groups (severe vs. comparison, moderate vs. comparison, mild vs. comparison, severe vs. moderate, etc.). The significance level was set at *P* < 0.05, the cluster size was > 30 voxels [26], and family-wise error (FWE) correction was applied for multiple comparisons. The analyses were adjusted for age, sex, education level, body mass index (BMI), smoking status, smoking index and WMH scores.

The partial correlation analysis was as follows: 8 regions of interest (ROIs) (including the bilateral orbital part of the inferior frontal gyrus; the left superior frontal gyrus, the middle frontal gyrus, the medial orbital gyrus, the parahippocampal/fusiform gyrus, the supplementary motor cortex, and the right thalamus) were selected for clusters showing differences among the four groups, and the GM density (GMD) values were extracted from the GM maps of each individual. Partial correlations between GMD values and clinical variables (MoCA scores, FEV1 measurements, PaO_2_, and SaO_2_) were performed with sex, age, education, BMI, smoking status, smoking index and WMH scores as covariates. Statistical significance was defined as *P* < 0.05.

### Tract-based spatial statistics (TBSS) analysis

TBSS statistical analyses were performed to compare diffusion indices, including FA, mean diffusivity (MD), axial diffusivity (AD), and radial diffusivity (RD), using a permutation-based non-parametric statistical method (the randomized part of the Functional Magnetic Resonance Imaging of the Brain Software Library; http://fsl.fmrib.ox.ac.uk/fsl/fslwiki/FSL) [26]. First, an F-test was performed to identify the WM areas that differed among the four groups, after which post hoc analyses were carried out to identify specific differences between the severe, moderate, or mild COPD group and the comparison. The number of permutations was set at 5000. Analyses were adjusted for age, sex, education level, BMI, smoking status, smoking index and WMH scores. The resultant statistical maps had a threshold of *P* < 0.05 and were FWE corrected for multiple comparisons using the threshold-free cluster enhancement option, which controls the rate of type I errors.

The International Consortium of Brain Mapping DTI-81 WM labels atlas was used to identify WM tracts of interest, with a total of 14 ROIs selected, including the genu, body, and splenium of the corpus callosum; the bilateral anterior corona radiata; the bilateral superior corona radiata; the bilateral cingulum; the left superior and interior longitudinal fasciculus; and the bilateral internal and left external capsule. Partial correlation analyses between imaging parameters (MD, AD or RD) and clinical variables (MoCA scores, FEV1, PaO_2_, SaO_2_) were carried out with sex, age, education, BMI, smoking status, smoking index and WMH scores as covariates. Statistical significance was defined as *P* < 0.05.

## Results

### Physiological and behavioural findings

There were no differences in age, sex, BMI, smoking status, or education among the mild, moderate, and severe COPD groups and the comparison group (*P* > 0.05). However, the indices of lung function (FEV1%) and blood gas parameters (PaO_2_ and SaO_2_) showed significant differences (P < 0.05), and when lung function decreased, PaO_2_ and SaO_2_ decreased (Table 1).

Total MoCA scores differed significantly among the four groups (*P* < 0.05). The average cognitive function score of the COPD groups was < 26, meaning that the patients had mild cognitive impairment (normal ≥26). The MoCA items scores for visuospatial executive function, attention, abstraction, delayed recall, and orientation differed significantly among the four groups (P < 0.05). The differences between groups are shown in Table 2.

**Table 2:** MoCA total and subsection scores of the participants

Variables (score)	Comparison group (*n* = 31)	Mild COPD (*n* = 29)	Moderate COPD (*n* = 29)	Severe COPD (*n* = 26)	F value	*P* value
*MoCA total*	26.10 ± 1.32	24.21 ± 2.23*	22.14 ± 3.56**	21.84 ± 2.79	17.73	0.000
*MoCA item z-score #*						
Visuospatial executive function	0.03 ± 1.00	− 0.88 ± 1.56*	− 1.97 ± 1.84**	−2.36 ± 1.70**	14.22	0.000
Naming	− 0.13 ± 1.00	− 0.41 ± 1.72	− 1.17 ± 2.84	− 0.69 ± 2.04	1.78	0.155
Attention	−0.01 ± 1.00	−0.33 ± 1.30	− 1.19 ± 1.65**	−1.11 ± 1.27**	5.71	0.001
Verbal	0.01 ± 1.01	−0.30 ± 1.77	−0.67 ± 1.34	−1.06 ± 1.68	1.87	0.131
Abstraction	0.17 ± 1.00	−0.88 ± 1.96*	−1.14 ± 1.92*	−0.85 ± 1.45*	2.89	0.039
Delayed recall	0.00 ± 1.00	−0.49 ± 1.36	−1.04 ± 1.31*	−0.87 ± 0.86*	4.75	0.004
Orientation	−0.01 ± 1.00	−0.60 ± 2.45	−1.17 ± 2.42	−2.34 ± 3.23***	5.14	0.002

Note: Data are presented as the means ± standard deviations. The z-scores for the cognitive items were compared using one-way analysis of variance with post hoc tests. *P* values were calculated with analysis of variance

Abbreviations: *#*The z-score was expressed as (individual score of COPD group - mean score of the comparison group)/standard deviation of the comparison group. *Significantly different from controls (*P* < 0.05). **Significantly different from patients with mild COPD (*P* < 0.05). ***Significantly different from patients with moderate COPD (*P* < 0.05)

### Change in GMD

Compared to the comparison group, COPD patients showed widespread GM atrophy in the bilateral orbital part of the inferior frontal gyrus; the left superior frontal gyrus, middle frontal gyrus, medial orbital gyrus, parahippocampal/fusiform gyrus, and supplementary motor cortex; and right thalamus (*P* < 0.05, FWE corrected; Additional file 1: Table S1).

**Fig. 1:**
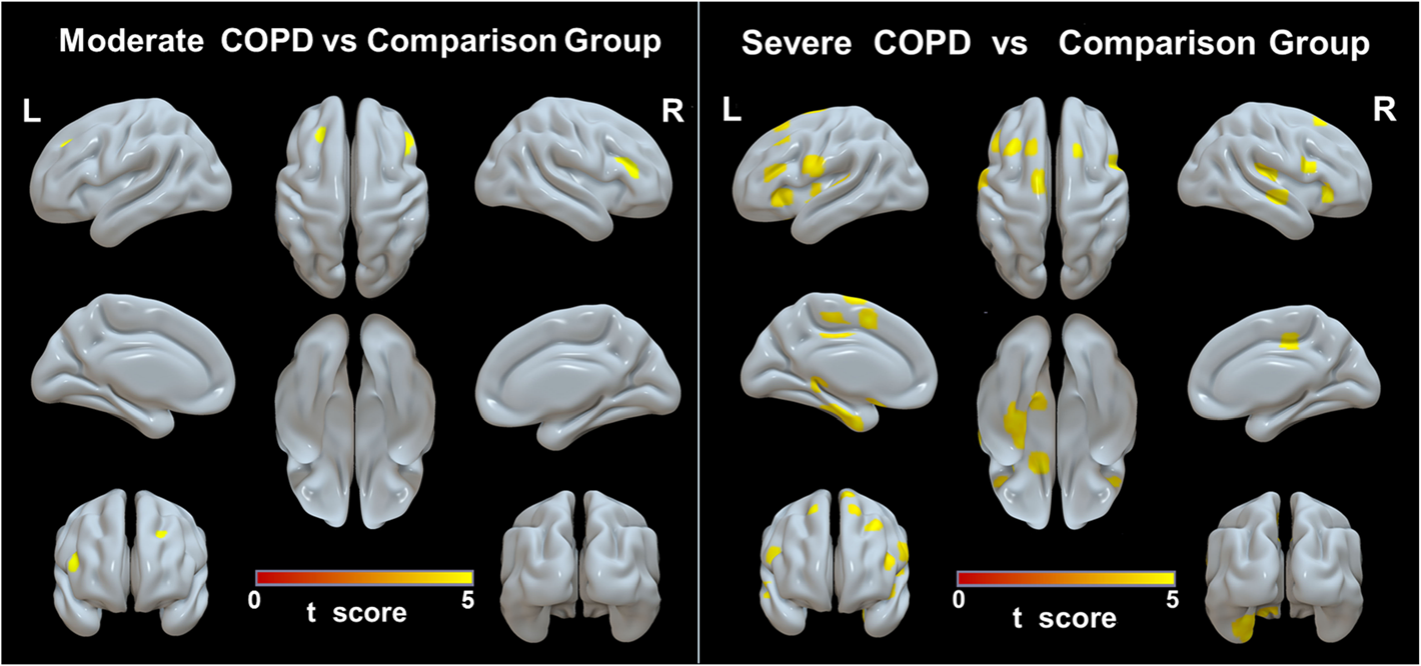
VBM results identifying atrophied brain areas in moderate and severe COPD patients compared to the comparison group (*P* < 0.05, FWE corrected) **Abbreviations**: *VBM* Voxel-based morphometry, *COPD* chronic obstructive pulmonary disease; and NCs, normal control

The number of brain regions with reduced GMD was not significantly different between mild COPD patients and the comparison group. Only a few brain regions (including the left middle frontal gyrus and right opercular part/triangular part of the inferior frontal gyrus) had reduced GMD in moderate COPD patients relative to the comparison group (*P* < 0.05, FWE corrected; Fig. 1 and Additional file 1: Table S2). Brain regions with reduced GMD relative to the comparison group were more extensive in patients with severe COPD than in those with moderate COPD; multiple brain regions demonstrated atrophy in the severe COPD group. The details of these results are shown in Fig. 1 and Additional file 1: Table S2 (*P* < 0.05, FWE corrected).

### Change in WM

Significant differences in MD, AD and RD were observed among the four groups, including changes in WM integrity in the corpus callosum, cingulum, fornix, corona radiata, posterior thalamic radiation, internal capsule, external capsule (left), superior and inferior longitudinal fasciculus (left) and inferior fronto-occipital fasciculus (left); the FA values were not significantly different among the four groups (P < 0.05, FWE corrected; Additional file 2: Fig. S1).

**Fig. 2:**
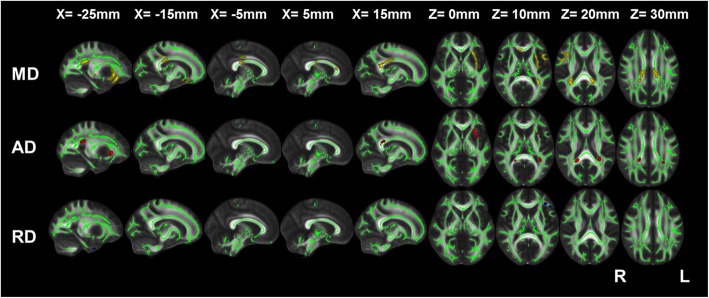
TBSS results showing the differences in diffusion indices between moderate COPD patients and the comparison group **Note**: Green represents the mean WM skeleton of all subjects; yellow, red and blue represent regions with increased MD, increased AD and increased RD, respectively, in moderate COPD patients (*P* < 0.05, FWE corrected). **Abbreviations**: TBSS, tract-based spatial statistics; COPD, chronic obstructive pulmonary disease; NCs, normal controls; WM, white matter; MD, mean diffusivity; AD, axial diffusivity; and RD, radial diffusivity

The increases in MD, AD, and RD in mild COPD patients relative to the comparison group were not statistically significant. Moderate COPD patients showed increases in MD and AD in the splenium of the corpus callosum, left posterior corona radiata and external capsule relative to the comparison group. Additional increases in MD were detected in the right superior corona radiata, genu and body of the corpus callosum; the left posterior thalamic radiation; and the bilateral cingulum (cingulate gyrus). RD was observed in the left longitudinal fasciculus (Fig. 2).

**Fig. 3:**
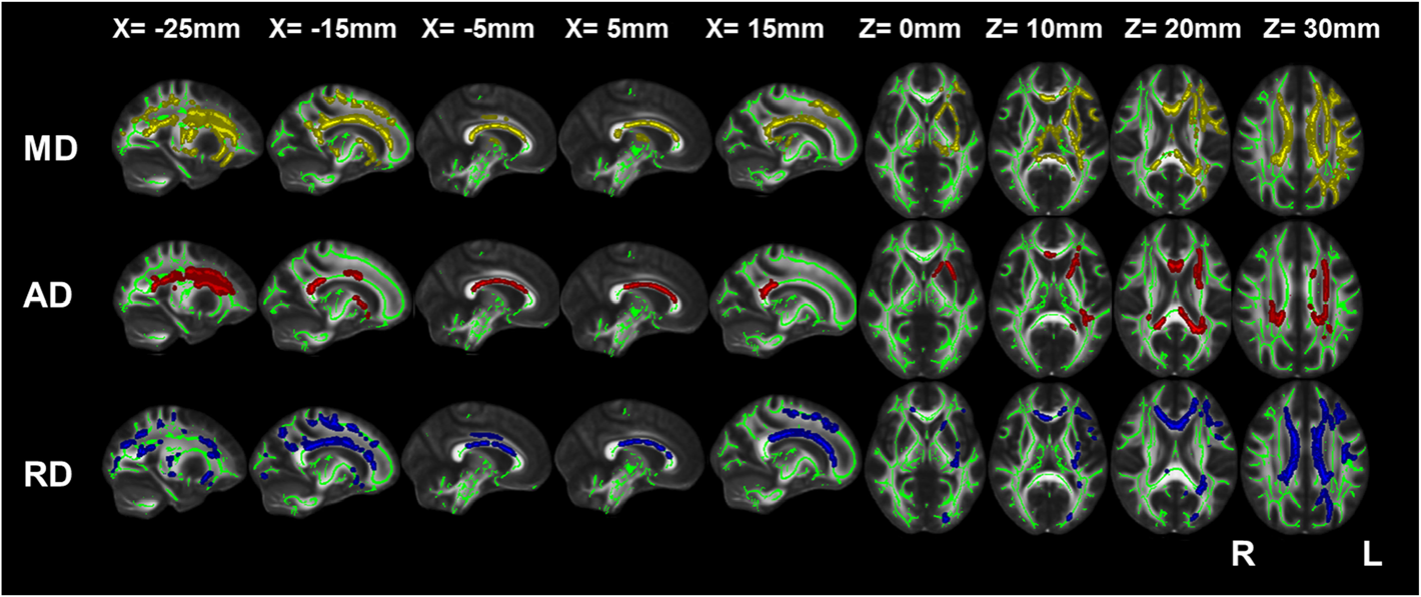
TBSS results of the differences in diffusion indices between severe COPD patients and the comparison group **Note**: Green represents the mean WM skeleton of all subjects; yellow, red and blue represent regions with increased MD, increased AD, and increased RD, respectively, in severe COPD patients (P < 0.05, FWE corrected). **Abbreviations**: TBSS, tract-based spatial statistics; COPD, chronic obstructive pulmonary disease; NCs, normal controls; WM, white matter; MD, mean diffusivity; AD, axial diffusivity; and RD, radial diffusivity

Relative to the comparison group, the severe COPD patients showed the most extensive changes in WM integrity, including increased MD, AD and RD values in the bilateral posterior corona radiate; the left anterior and superior corona radiata, left cingulum (cingulate gyrus); the left posterior thalamic radiation; the left anterior limb of the internal capsule; the left external capsule; and the genu, body and splenium of the corpus callosum. Additional increases in MD or RD were observed in the fornix, bilateral cingulum (cingulate gyrus), right anterior and superior corona radiata, left posterior limb of the internal capsule, left superior and inferior longitudinal fasciculus, and left inferior fronto-occipital fasciculus (Fig. 3).

### Correlational analysis

There was no statistically significant correlation between GMD or WM changes and PaO_2_ or SaO_2_.

**Fig. 4:**
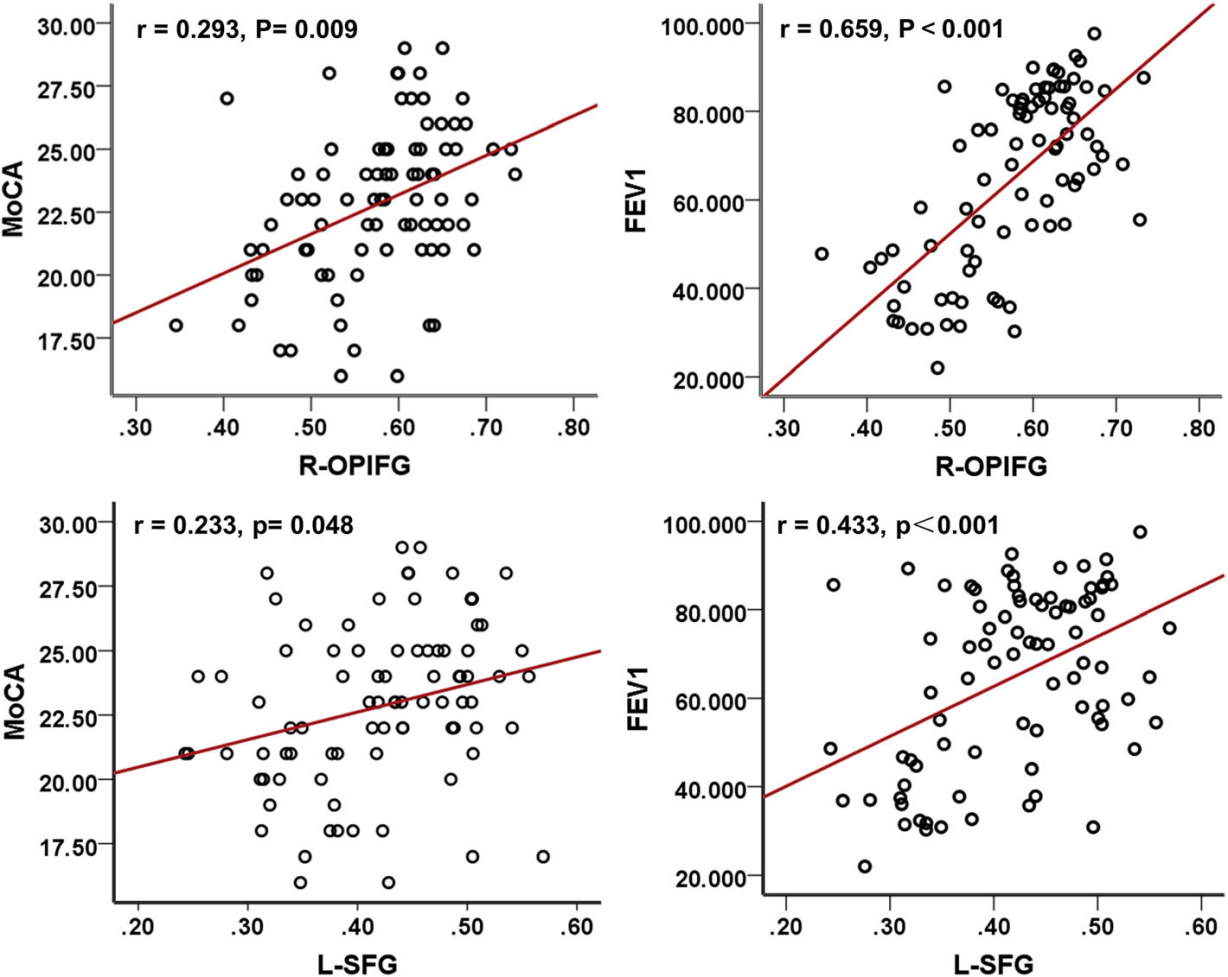
Correlation analyses among MoCA scores, FEV1, and GMD in COPD patients. **Abbreviations**: MoCA, Montreal Cognitive Assessment; FEV1, forced expiratory volume in 1 s; COPD, chronic obstructive pulmonary disease; R-OPIFG, right orbital part of the inferior frontal gyrus; and L-SFG, left superior frontal gyrus

In the COPD group, the GMDs in the left superior frontal gyrus and right orbital part of the inferior frontal gyrus were positively correlated with MoCA scores (r = 0.233, *P* = 0.048; r = 0.293, *P* = 0.009, respectively) and FEV1 values (r = 0.433, P<0.001; r = 0.659, P<0.001; Table 3 and Fig. 4). The MD and RD values of the body of the corpus callosum and the AD value of the bilateral superior corona radiata were negatively correlated with FEV1 and MoCA scores (Table 3 and Additional file 1: Table S3).

**Table 3:** Correlations of imaging parameters with MoCA scores and FEV1 in COPD

Brain regions	MoCA		FEV1	
	r	*P*	r	*P*
GMD				
Left superior frontal gyrus	0.233	0.048	0.433	<0.001
Left middle frontal gyrus	0.058	0.616	0.447	<0.001
Left medial orbital gyrus	0.151	0.186	0.600	<0.001
Left pars orbitalis of the inferior frontal gyrus	0.154	0.179	0.544	<0.001
Right orbital part of the inferior frontal gyrus	0.293	0.009	0.659	<0.001
Left supplementary motor cortex	0.105	0.359	0.615	<0.001
Left parahippocampal / fusiform gyrus	0.178	0.119	0.553	<0.001
Right thalamus	0.078	0.495	0.116	0.312
MD				
Corpus callosum (body)	−0.232	0.039	− 0.324	0.004
AD				
Left superior corona radiata	−0.249	0.027	−0.322	0.004
Right superior corona radiata	−0.300	0.007	−0.271	0.016
RD				
Corpus callosum (body)	−0.264	0.019	−0.311	0.005

**Abbreviations**: MoCA, Montreal Cognitive Assessment; FEV1, forced expiratory volume in 1 s; GMD, grey matter density; COPD, chronic obstructive pulmonary disease; MD, mean diffusivity; AD, axial diffusivity; RD, radial diffusivity

## Discussion

In this study, we found that the MoCA scores of patients with COPD were gradually reduced from mild to severe COPD. Moreover, the brain structures of the abovementioned groups of COPD patients showed a trend of progressive change.

We found that COPD patients’ scores on MoCA items that measure aspects of executive function, attention and delayed memory were lower than the comparison group, similar to the results of a previous study demonstrating that irreversible restriction of airflow in COPD patients may result in a reduced oxygen supply, which may cause damage to neurons in the brain, and continuous hypoxia may damage people’s delayed recall and attention [27]. Incalzi et al. [28] also found that COPD patients with hypoxia and high carbon dioxide levels had a characteristic pattern of cognitive decline characterized by disorders of executive function and attention. The literature has demonstrated the presence of mild cognitive impairment (MCI) in patients with obstructive sleep apnoea-hypopnoea syndrome (OSAHS); evaluation of MoCA items further revealed selective reductions in visuospatial skills, executive function, attention and delayed memory, and MoCA scores were correlated significantly with serum levels of TNF-α [29]. Crisan et al. [30] found that low FEV1 was associated with decreased MoCA scores. These results support our findings and suggest that the cognitive impairment accompanying COPD may be caused by hypoxia, carbon dioxide retention, inflammatory factors or pulmonary dysfunction.

Relative to the comparison group, the GMD of patients with COPD was mainly reduced in the prefrontal cortex, limbic system structures and thalamus, which was partially consistent with the findings of Zhang et al. [15] and similar to results from patients with sleep apnoea syndrome [31–33]. We found that the GMD was decreased in the left superior frontal gyrus, left orbital part of the inferior frontal gyrus, left medial orbital gyrus, and left middle frontal gyrus, which mainly participate in high-level cognitive functions (e.g., visual motion synchronization, language fluency, and executive functions) [34]. The dorsal cortex of the prefrontal cortex and the dorsal prefrontal cortex of the frontoparietal network were obviously atrophied, which may lead to declines in visual memory and reconstruction in COPD patients [16]. We also observed that the GMD in the limbic system (parahippocampal gyrus) was decreased in COPD patients. The parahippocampal gyrus is a key area of the limbic system that plays an important role in the regulation of emotion, motivation and memory [35, 36]. Limbic structures are connected to the frontal and temporal lobes and have subcortical connections that are critical for cognition and memory [37]. Thus, GM damage in these areas may lead to impairments in cognition and memory. The thalamus plays an important role in the Papez circuit and is connected to the medial temporal lobe [38], which is essential for episodic memory [39]. GM damage to the thalamus can affect cognitive memory functions. Abnormal neuronal activity in the prefrontal-limbic network, including the dorsal prefrontal and orbitofrontal cortices and the parahippocampal gyrus, is associated with a decrease in working memory [40].

FA is sensitive to microstructural changes; MD is a measure of total diffusion within a voxel [41]; AD increases in WM tracts with brain maturation and is sensitive to axonal injury; and RD is sensitive to axonal diameters and demyelination [42]. Thus, a significant decrease in FA and increases in AD and RD indicate the possibility of injury to axons and/or myelin [41, 43]. However, we did not find any significant difference in FA values among the four groups. FA is affected by MD, AD and RD [44]. However, when the values of MD, AD and RD increase simultaneously, the FA value may not change, which was observed in our research.

Therefore, in this study, we combined these three values to observe changes in WM microstructure in COPD patients. We found that WM changes in COPD patients were localized mainly in the corona radiata, cingulate gyrus, corpus callosum and superior and inferior longitudinal fasciculus, and the changes in the severe group were more extensive than those in the moderate group. WM changed more broadly than GM. Although WM, which occupies 50% of the total brain volume in humans, has a metabolic rate similar to that of GM [45, 46], WM receives a disproportionately small blood supply and little collateral circulation, making it particularly susceptible to ischaemic insults [46, 47], in which chronic systemic inflammation, tissue hypoxia and oxidative stress play crucial roles [48]. Axonal changes interfere with the communication between brain structures and thus alter the functions of these structures [49].

We found that the GMD of the left superior frontal gyrus and right orbital part of the inferior frontal gyrus, the MD and RD of the body of the corpus callosum and the AD of the bilateral superior corona radiata were correlated with FEV1 and MoCA scores. Previous studies showed that the left side of the superior frontal cortex exhibited greater thinning [50] and that there was impaired functional connectivity with the bilateral inferior frontal gyrus in obstructive sleep apnoea [51]. The superior corona radiata, an associated fibre tract in the prefrontal cortex, is connected to the internal capsule. The body of the corpus callosum comprises commissural fibres that connect the bilateral cerebral hemispheres. All three of the aforementioned fibre tracts are involved in cognitive function in COPD patients. We speculate that persistent reductions in lung function may lead to atrophy of the left superior frontal gyrus and right orbital part of the inferior frontal gyrus as well as WM changes in the body of the corpus callosum and bilateral superior corona radiata, ultimately resulting in cognitive impairment.

However, we did not find a clear correlation between PaO_2_ or SaO_2_ and brain structural changes in COPD patients, suggesting that hypoxia may not be the main mechanism of brain structural changes and cognitive impairment in this disease. The brain pathological mechanism of cognitive impairment in COPD patients may be very complex. Savchenko et al. and Sakurai et al. found that FEV1 was negatively correlated with the systemic inflammatory factor IL-26 [52], a biomarker of inflammation. The neutrophil-to-lymphocyte ratio (NLR) is associated with the severity of COPD [53]. Inflammatory factor ‘spill-over’ [54] may cause cognitive impairment in COPD patients. Wang et al. [55] studied brain activity in stable patients with COPD and found that the mean signal values in the cluster with reduced amplitude of low-frequency fluctuation (ALFF) were significantly negatively correlated with PaCO_2_. Inflammatory factors or carbon dioxide retention may be potential mechanisms that merit further attention in future research.

### Limitations

This study had some limitations. First, we did not include comprehensive neuropsychological assessments of the comparison and COPD groups; this omission may have introduced some bias in the classification of groups. Second, we used only the MoCA to assess the patients’ cognitive status; the items included on the MoCA are limited in their ability to measure subdomains of cognition. In the future, comprehensive neuropsychological assessment could be used to fully evaluate patients’ cognition. Third, patients with COPD often have more additional medical problems, which we listed an exclusion criterion. The exclusion of patients with comorbidities can limit the representativeness of the sample; however, we used a long scan time, and COPD patients with other severe illnesses may have faced discomfort if they had participated. Fourth, the study was cross-sectional and showed that lung function in COPD patients was related to changes in brain structure and cognitive function. Previous studies have shown that long-term oxygen therapy and exercise can improve lung and, to some extent, cognitive function [27]. A longitudinal study examining the role of pulmonary function as an independent factor in COPD is planned for the future. Fifth, patients with COPD used steroids to relieve symptoms during an acute attack. Although studies [56] have shown that steroids cause changes in brain structure, they are necessary for humane patient care. Finally, we did not perform multiple comparison correction for the correlation analyses between imaging parameters and MoCA and FEV1. However, our study is exploratory in nature, and the results showed a modest strength of the association that cannot pass the multiple comparison correction. In the future, we would increase the sample size and make more stringent adjustment.

## Conclusions

These findings suggest that patients with COPD exhibit progressive structural impairments in both GM and WM, along with impaired levels of lung function, highlighting the importance of early clinical interventions.

## Supplementary information

**Additional file 1: Table S1.** Brain areas with significant inter-group differences in GMD according to analysis of variance. **Table S2.** Brain areas with significant GMD decreases in severe and moderate COPD patients. **Table S3.** Correlation analysis between WM parameters and (MoCA scores and FEV1) in COPD.

**Additional file 2: Figure S1. **TBSS results of diffusion indices in the four groups. Note: Extensive WM differences were observed among the four groups in various brain regions. Green represents the mean WM skeleton of all subjects; yellow, red and blue represent regions with significantly different MD, AD, and RD, respectively (*P* < 0.05, FWE corrected). Abbreviations: TBSS, tract-based spatial statistics; WM, white matter; MD, mean diffusivity; AD, axial diffusivity; and RD, radial diffusivity.

## Data Availability

The datasets analysed during the current study are available from the corresponding author on reasonable request.

## Abbreviations

AD: axial diffusivity
ANOVA: analysis of variance.
COPD: chronic obstructive pulmonary disease
DTI: diffusion tensor imaging
FA: fractional anisotropy
FEV1: forced expiratory volume in 1 s
GMD: grey matter density
MD: mean diffusivity
MoCA: Montreal Cognitive Assessment
RD: radial diffusivity
TBSS: tract-based spatial statistics
VBM: voxel-based morphometry
WM: white matter
