# Supplementary material for: Patterns of brain structural alteration in COPD with different levels of pulmonary function impairment and its association with cognitive deficits
Source: BMC Pulm Med. 2019 Nov 7;19:203. doi: 10.1186/s12890-019-0955-y (PMC6839173; doi:10.1186/s12890-019-0955-y)
Supplement: Supplementary file 1 — Additional file 1: Table S1. Brain areas with significant inter-group differences in GMD according to analysis of variance. Table S2. Brain areas with significant GMD decreases in severe and moderate COPD patients. Table S3. Correlation analysis between WM parameters and (MoCA scores and FEV1) in COPD. [file 12890_2019_955_MOESM1_ESM.doc]

**Supplementary materials:** The imaging protocol.

The imaging protocol consisted of the following: (1) a T1-weighted, three-dimensional (3D) structure spoiled gradient recalled echo sequence (repetition time [TR]/echo time [TE]/inversion time: 8.5/3.3/900 ms; matrix: 256 × 256; FOV: 240 × 240 mm; slice thickness: 1.2 mm without intervals; and scanning time: 8 min 59 s); (2) an axial T2-weighted DTI spin echo single-shot echo planar imaging sequence (TR/TE/number of excitations: 10,000 ms/87 ms/2; matrix: 128 × 130; FOV: 240 × 240 mm; section thickness: 4 mm without intervals; maximum b value: 1000 s/mm^2^; 30 non-collinear diffusion directions; and scanning time: 10 min 30 s); and (3) axial T2-weighted (TR/TE: 5000/120 ms; matrix: 384 × 256; FOV: 240 × 240 mm; slice thickness: 5 mm; spacing: 1.5 mm; and scanning time: 1 min 06 s) and fluid-attenuated inversion recovery images sequence (TR/TE: 9000/120 ms; matrix: 384 × 256; FOV: 240 × 240 mm; slice thickness: 5 mm; spacing: 1.5 mm; and scanning time: 1 min 57 s).

| **Supplementary Table 1.** Brain areas with significant inter-group differences in GMD according to analysis of variance. | | | | | | | | | | | | | | | | |
| --- | --- | --- | --- | --- | --- | --- | --- | --- | --- | --- | --- | --- | --- | --- | --- | --- |
| Area | | Hemisphere | Cluster size (voxels) | | Z | | *P* | | MNI coordinates | | | | | | | BA |
|  |  |  |  |  |  |  |  |  | X | | | Y | | Z | |  |
| Superior frontal gyrus | | L | 80 | | 5.70 | | 0.001 | | -10 | | | -4 | | 72 | | 6 |
| Middle frontal gyrus | | L | 74 | | 5.67 | | 0.001 | | -38 | | | 24 | | 50 | | 9 |
| Orbital part of the inferior frontal gyrus | | L | 91 | | 5.68 | | 0.001 | | -40 | | | 22 | | -3 | | 47 |
|  |  | R | 51 | | 6.03 | | 0.000 | | 42 | | | 27 | | -4 | | 47 |
| Medial orbital gyrus | | L | 70 | | 5.87 | | 0.000 | | -14 | | | 14 | | -21 | | 11 |
| SMC | | L | 42 | | 5.72 | | 0.001 | | -3 | | | 8 | | 51 | | 32 |
| PHG/FG | | L | 304 | | 6.19 | | 0.000 | | -30 | | | -24 | | -28 | | 20 |
|  | |  |  | | 6.19 | | 0.000 | | -20 | | | -15 | | -30 | | 36 |
|  | |  |  | | 6.01 | | 0.000 | | -27 | | | -4 | | -40 | | 20 |
| Thalamus | | R | 77 | | 5.15 | | 0.014 | | 10 | | | -12 | | 6 | | - |
| **Note**: P < 0.05 (FWE corrected), Cluster size > 30 voxels.  **Abbreviations**: GMD, grey matter density; COPD, chronic obstructive pulmonary disease; BA, Brodmann’s area; FG, fusiform gyrus; PHG, parahippocampal gyrus; SMC, supplementary motor cortex. | | | | | | | | | | | | | | | | |
| **Supplementary Table 2.** Brain areas with significant GMD decreases in severe and moderate COPD patients. | | | | | | | | | | | | | | | | |
| Area | Hemisphere | | | Cluster size (voxels) | | Z | | *P* | | MNI coordinates | | | | | BA | |
|  |  |  |  |  |  |  |  |  |  | X | Y | | Z | |  |  |
| **Severe COPD patients vs.Comparison group** | | | | | | | | | | | | | | | | |
| Medial orbital gyrus | L | | | 86 | | 6.14 | | 0.000 | | -14 | 15 | | -21 | | 11 | |
| Superior frontal gyrus | L | | | 48 | | 5.61 | | 0.001 | | -20 | 24 | | 62 | | 8 | |
|  | R | | | 33 | | 5.72 | | 0.001 | | 21 | 21 | | 60 | | 8 | |
| Middle frontal gyrus | L | | | 103 | | 5.95 | | 0.000 | | -36 | 24 | | 48 | | 9 | |
| OPIFG | R | | | 38 | | 5.52 | | 0.002 | | 52 | 10 | | 21 | | 44 | |
| TPIFG | L | | | 48 | | 5.62 | | 0.001 | | -45 | 27 | | 20 | | 48 | |
| Orbital part of the inferior frontal gyrus | L | | | 249 | | 6.32 | | 0.000 | | -40 | 22 | | -3 | | 47 | |
|  | R | | | 116 | | 6.27 | | 0.000 | | 42 | 27 | | -4 | | 47 | |
| Superior temporal gyrus | L | | | 79 | | 5.17 | | 0.010 | | -56 | -26 | | 12 | | 48 | |
|  | R | | | 49 | | 5.07 | | 0.016 | | 38 | -24 | | 15 | | 48 | |
|  |  |  |  | 56 | | 5.36 | | 0.004 | | 54 | -15 | | -8 | | 22 | |
| Insula | L | | | 33 | | 5.23 | | 0.007 | | -45 | 0 | | -2 | | 48 | |
| Postcentral gyrus | L | | | 103 | | 5.76 | | 0.001 | | -56 | -8 | | 21 | | 43 | |
| SMC | L | | | 96 | | 6.36 | | 0.000 | | -3 | 8 | | 51 | | 32 | |
|  |  | | | 147 | | 6.36 | | 0.000 | | -10 | -3 | | 70 | | 6 | |
| Lingual gyrus | L | | | 52 | | 5.47 | | 0.002 | | -15 | -33 | | -2 | | 27 | |
| MCG | L | | | 200 | | 5.52 | | 0.002 | | -3 | -22 | | 51 | | 23 | |
|  |  | | |  | | 5.48 | | 0.002 | | -3 | -15 | | 44 | | 23 | |
|  | R | | |  | | 5.30 | | 0.005 | | 8 | -24 | | 45 | | 23 | |
| PHG/FG | L | | | 591 | | 6.84 | | 0.000 | | -20 | -15 | | -30 | | 36 | |
|  |  | | |  | | 6.74 | | 0.000 | | -30 | -24 | | -28 | | 20 | |
|  |  | | |  | | 6.39 | | 0.000 | | -27 | -4 | | -40 | | 20 | |
| **Moderate COPD patients vs. Comparison group** | | | | | | | | | | | | | | | | |
| Middle frontal gyrus | L | | | 51 | | 5.59 | | 0.001 | | -24 | 28 | | 34 | | 9 | |
| TPIFG/OPIFG | R | | | 31 | | 5.17 | | 0.010 | | 45 | 30 | | 15 | | 45 | |
|  |  | | |  | | 5.06 | | 0.017 | | 42 | 22 | | 21 | | 48 | |
| **Note**: Cluster size > 30 voxels; P < 0.05 (FWE corrected).  **Abbreviations**: GMD, grey matter density; COPD, chronic obstructive pulmonary disease; NCs, normal controls; ACG, anterior cingulate gyrus; BA, Brodmann’s area; FG, fusiform gyrus; MCG, middle cingulate gyrus; OPIFG, opercular part of the inferior frontal gyrus; PHG, parahippocampal gyrus; SMC, supplementary motor cortex; TPIFG, triangular part of the inferior frontal gyrus.   | **Supplementary Table 3**. Correlation analysis between WM parameters and (MoCA scores and FEV1) in COPD | | | | | | | | | | | --- | --- | --- | --- | --- | --- | --- | --- | --- | --- | | Brain regions | **MoCA** | | | | **FEV1** | | | | | | **r** | | ***P*** | | **r** | | | ***P*** | | | **MD** | | | | | | | | | | | Corpus callosum (genu) | | -0.130 | | 0.253 | | -0.094 | | 0.411 | | | Corpus callosum (body) | | -0.232 | | 0.039 | | -0.324 | | 0.004 | | | Corpus callosum (splenium) | | -0.069 | | 0.545 | | -0.224 | | 0.047 | | | Left anterior corona radiata | | -0.273 | | 0.015 | | -0.145 | | 0.203 | | | Right anterior corona radiata | | -0.266 | | 0.018 | | -0.188 | | 0.096 | | | Left superior corona radiata | | -0.294 | | 0.009 | | -0.202 | | 0.074 | | | Right superior corona radiata | | -0.320 | | 0.004 | | -0.165 | | 0.146 | | | Left cingulum | | -0.125 | | 0.273 | | -0.168 | | 0.139 | | | Right cingulum | | -0.167 | | 0.142 | | -0.169 | | 0.135 | | | Left superior longitudinal fasciculus | | -0.230 | | 0.042 | | -0.215 | | 0.057 | | | Left interior longitudinal fasciculus | | -0.239 | | 0.034 | | -0.149 | | 0.191 | | | Left internal capsule | | -0.307 | | 0.006 | | -0.088 | | 0.439 | | | Right internal capsule | | -0.191 | | 0.092 | | -0.069 | | 0.547 | | | Left external capsule | | -0.336 | | 0.002 | | -0.081 | | 0.479 | | | **AD** | | | | | | | | | | | Corpus callosum (genu) | | -0.001 | | 0.994 | | -0.202 | | 0.074 | | | Corpus callosum (body) | | -0.006 | | 0.961 | | -0.180 | | 0.113 | | | Corpus callosum (splenium) | | -0.007 | | 0.949 | | -0.263 | | 0.019 | | | Left anterior corona radiata | | -0.234 | | 0.038 | | -0.221 | | 0.051 | | | Right anterior corona radiata | | -0.183 | | 0.107 | | -0.254 | | 0.024 | | | Left superior corona radiata | | -0.249 | | 0.027 | | -0.322 | 0.004 | | | | Right superior corona radiata | | -0.300 | | 0.007 | | -0.271 | 0.016 | | | | Left cingulum | | -0.056 | | 0.622 | | -0.179 | | | 0.115 | | Right cingulum | | -0.079 | | 0.490 | | -0.167 | | | 0.142 | | Left superior longitudinal fasciculus | | -0.239 | | 0.034 | | -0.093 | | | 0.414 | | Left interior longitudinal fasciculus | | -0.188 | | 0.096 | | -0.054 | | | 0.638 | | Left internal capsule | | -0.263 | | 0.019 | | -0.119 | | | 0.296 | | Right internal capsule | | -0.290 | | 0.010 | | -0.069 | | | 0.548 | | Left external capsule | | -0.318 | | 0.004 | | -0.095 | | | 0.405 | | **RD** | | | | | | | | | | | Corpus callosum (genu) | | -0.157 | | 0.166 | | -0.041 | | | 0.721 | | Corpus callosum (body) | | -0.264 | | 0.019 | | -0.311 | | | 0.005 | | Corpus callosum(splenium) | | -0.089 | | 0.434 | | -0.156 | | | 0.169 | | Left anterior corona radiata | | -0.261 | | 0.020 | | -0.100 | | | 0.378 | | Right anterior corona radiata | | -0.270 | | 0.016 | | -0.143 | | | 0.209 | | Left superior corona radiata | | -0.295 | | 0.008 | | -0.111 | | | 0.320 | | Right superior corona radiata | | -0.286 | | 0.011 | | -0.079 | | | 0.491 | | Left cingulum | | -0.136 | | 0.231 | | -0.121 | | | 0.289 | | Right cingulum | | -0.164 | | 0.148 | | -0.114 | | | 0.317 | | Left superior longitudinal fasciculus | | -0.170 | | 0.135 | | -0.134 | | | 0.239 | | Left interior longitudinal fasciculus | | -0.223 | | 0.048 | | -0.171 | | | 0.132 | | Left internal capsule | | -0.325 | | 0.003 | | -0.083 | | | 0.470 | | Right internal capsule | | -0.311 | | 0.005 | | -0.070 | | | 0.538 | | Left external capsule | | -0.324 | | 0.004 | | -0.070 | | | 0.541 | | **Abbreviations**: WM, white matter; MoCA, Montreal Cognitive Assessment; FEV1, forced expiratory volume in 1 s; COPD, chronic obstructive pulmonary disease; MD, mean diffusivity; AD, axial diffusivity; RD, radial diffusivity. | | | | | | | | | | | | | | | | | | | | | | | | | | |
