# Supplementary figures and images for: Patterns of brain structural alteration in COPD with different levels of pulmonary function impairment and its association with cognitive deficits
Source: BMC Pulm Med. 2019 Nov 7;19:203. doi: 10.1186/s12890-019-0955-y (PMC6839173; doi:10.1186/s12890-019-0955-y)

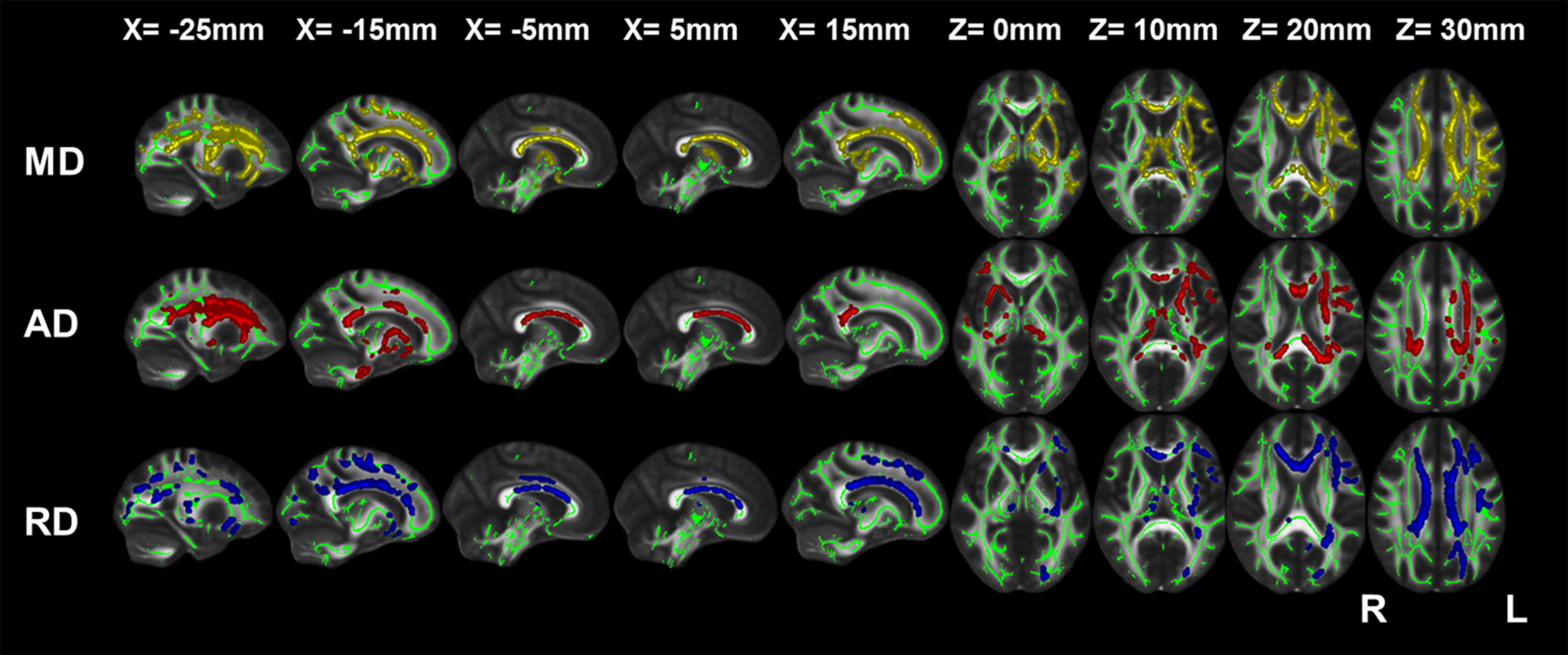

Supplement: Supplementary file 2 — Additional file 2: Figure S1. TBSS results of diffusion indices in the four groups. Note: Extensive WM differences were observed among the four groups in various brain regions. Green represents the mean WM skeleton of all subjects; yellow, red and blue represent regions with significantly different MD, AD, and RD, respectively (P < 0.05, FWE corrected). Abbreviations: TBSS, tract-based spatial statistics; WM, white matter; MD, mean diffusivity; AD, axial diffusivity; and RD, radial diffusivity. [file 12890_2019_955_MOESM2_ESM.jpg]
